# Tetraphenylethylene-Based Photoluminescent Self-Assembled Nanoparticles: Preparation and Biological Evaluation

**DOI:** 10.1021/acsmedchemlett.3c00396

**Published:** 2023-09-29

**Authors:** Eleonora Colombo, Elif Merve Aydın, İdil Su Canıtez, Laura Polito, Marta Penconi, Alberto Bossi, Elisa Impresari, Daniele Passarella, Sabrina Dallavalle, Constantinos M. Athanassopoulos, Sara Pellegrino, Irem Durmaz Şahin, Michael S. Christodoulou

**Affiliations:** † Dipartimento di Chimica, Universitá degli Studi di Milano, 20133 Milano, Italy; ‡ Ann Romney Center for Neurologic Diseases, Department of Neurology, Brigham and Women’s Hospital and Harvard Medical School, Boston, Massachusetts 02115, United States; § Koc University Research Center for Translational Medicine (KUTTAM), Sariyer, Istanbul 34450, Turkey; ⊥ Istituto di Scienze e Tecnologie Chimiche “Giulio Natta”, SCITEC−CNR, 20138 Milano, Italy; ¶ Istituto di Scienze e Tecnologie Chimiche “Giulio Natta”, SCITEC−CNR, 20138, Milano, Italy; # SmartMatLab Center, 20133 Milano, Italy; ∥ DISFARM, Dipartimento di Scienze Farmaceutiche, Sezione Chimica Generale e Organica “A. Marchesini”, Università degli Studi di Milano, 20133 Milano, Italy; △ School of Medicine, Koc University, Sariyer, Istanbul 34450, Turkey; □ Department of Food, Environmental and Nutritional Sciences (DeFENS), University of Milan, via Celoria 2, 20133 Milan, Italy; ■ Synthetic Organic Chemistry Laboratory, Department of Chemistry, University of Patras, GR-26504 Patras, Greece

**Keywords:** Tetraphenylethylene, self-assembled nanoparticles, cancer cell lines, podophyllotoxin, *N*-desacetylthiocolchicine, cabazitaxel, aggregation-induced emission

## Abstract

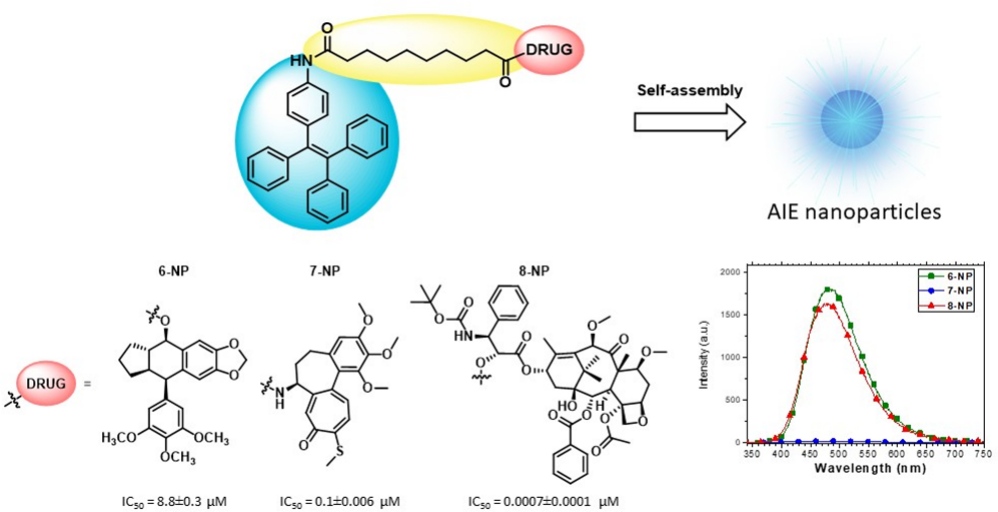

The conjugation of tetraphenylethylene (TPE) with podophyllotoxin, *N*-desacetylthiocolchicine, and cabazitaxel through a sebacic acid linker led to the formation of fluorescent nanoparticles. Dynamic light scattering (DLS) and photoluminescence spectroscopy were used for the identification and characterization of the fluorescent nanoparticles. The biological evaluation was determined in three human ovarian (KURAMOCHI, OVCAR3, OVSAHO) and three human breast (MCF7, SKBR 3, and MDA-MB231) cancer cell lines. In the case of cabazitaxel, the nanoparticles maintained the activity of the parent drug, at the low nanomolar range, while exhibiting high blue fluorescence. The internalization of the fluorescent NPs into cells was detected using immunofluorescence assay.

In recent years, nanomedicine has become increasingly more important as a tool to improve the bioavailability of drugs used for the treatment of various diseases, as it can help overcome a range of different issues including low bioavailability due to poor absorption and degradation after reaching the target site.^1^

Our group is particularly interested in a research niche in nanotechnology that is represented by self-assembling drug conjugates able to spontaneously form nanoparticles (NPs) in aqueous media. These conjugates present the same general structure in which the drug is covalently linked to lipophilic moieties, able to induce the structural organization of building blocks because of specific local interactions. This kind of NPs is easy to obtain and can reach high local drug concentrations in tissues, weakening the systemic toxicity of drugs. Over the years, we have reported different constructs, designing conjugates able to release the drug in cellular media,^2−4^ hetero-NPs bearing two different drugs,^5−7^ and fluorescent hetero-NPs.^5,8^ The fundamental moiety of this kind of NPs is the self-assembly inducer that could either be squalene,^2,5,6,8−11^ 4-(1,2-diphenylbut-1-en-1-yl) aniline,^3,12,13^ 20-hydroxyecdisone,^7^ betulinic acid,^14^ or cannabidiol.^4^ In all cases, the choice of self-assembly inducer is important for the formation of NPs.

We envisage using the 4-amino tetraphenylethylene (TPE) scaffold (**1**, Figure 1) as the self-assembly inducer, a compound similar to the previously mentioned 4-(1,2-diphenylbut-1-en-1-yl) aniline. Replacement of the ethyl chain with a phenyl ring provides a tetraphenylethylene (TPE) moiety, one of the most promising aggregation-induced emission (AIE) luminophores.^15^ AIE is a phenomenon correlated to certain materials that show negligible or extremely weak emission in dilute solution, while they brightly emit in the solid or aggregate state.^16^ Due to this interesting behavior, AIEgens have been increasingly applied in the design of materials involving such aggregation states, e.g., nanoparticles.^17^ Examples of AIE nanoparticles are present in literature, mainly as diagnostic tools^18,19^ but also for drug delivery because of their multiple functions including bioluminescence, drug release monitoring, and low biological toxicity.^17,20,21^ Recently, we showed that the conjugation of fatty acids with the 4-amino TPE leads to the formation of supramolecular aggregates that are able to self-assemble into different supramolecular emissive structures depending on the chemical composition and water content.^22^ Here, we exploited the same approach in order to obtain emissive self-assembling drug conjugates. This would make possible an easy preparation of fluorescent NPs, an important tool to follow the dynamics of drug internalization.^23,24^ For the synthesis of these NPs, three different drugs, podophyllotoxin (**3**), *N*-desacetylthiocolchicine (**4**), and cabazitaxel (**5**), were selected, all of them with the ability to interact with microtubules as stabilizers or destabilizers, connected with sebacic acid (**2**) as the linker^3,11^ (Figure 1).

**Figure 1:**
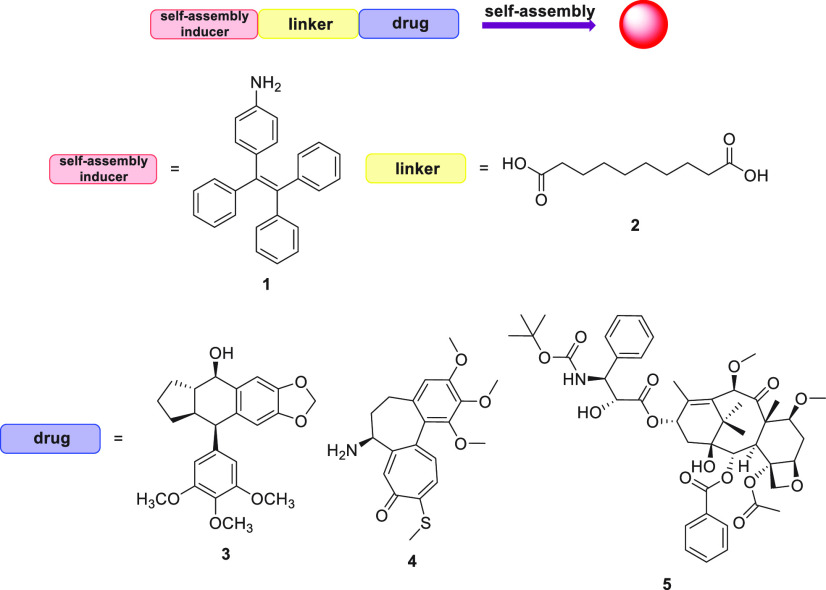
Building blocks of the conjugates.

Three different conjugates **6**, **7**, and **8** (Figure 2) were synthesized according to Schemes 1 and 2. A McMurry coupling^25,26^ between benzophenone (**9**) and 4-aminobenzophenone (**10**) furnished the self-assembly inducer 4-(1,2,2-triphenylvinyl) aniline (4-amino TPE) (**1**),^13^ which was then condensed with sebacic acid (**2**) to provide the desired intermediate acid **11** (Scheme 1).

**Figure 2:**
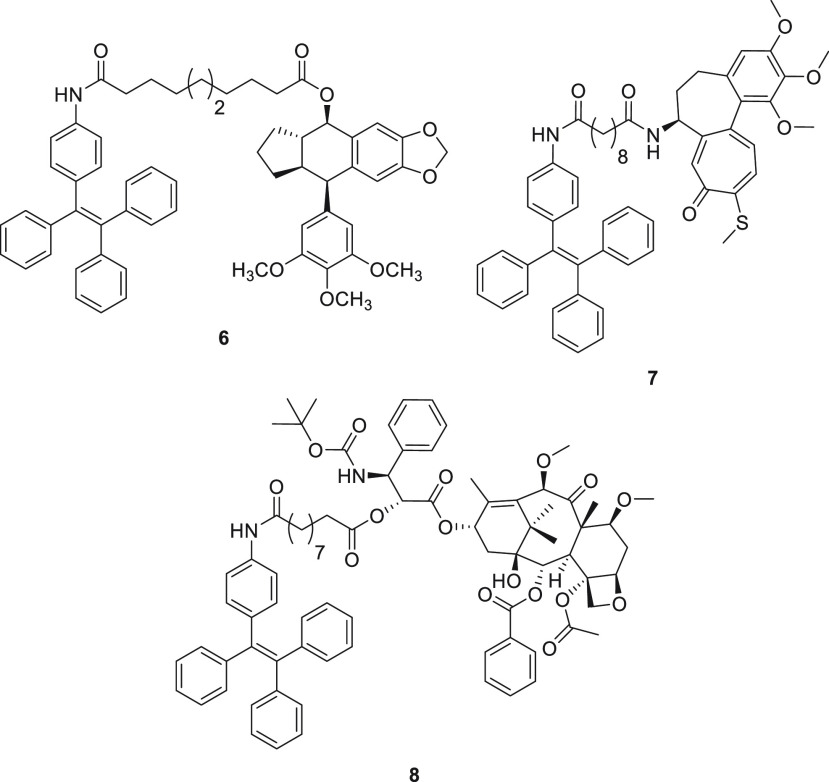
Structures of the obtained conjugates **6**, **7**, and **8**.

**Scheme 1:**
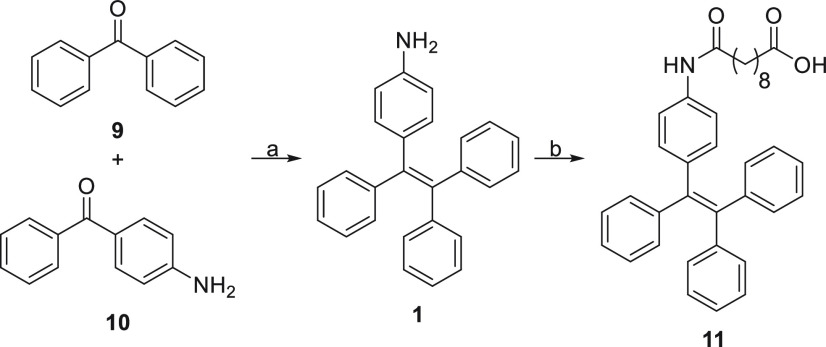
Synthesis of Intermediate Acid **11** Reagents and conditions: (a) Zn, TiCl_4_, CH_2_Cl_2_, −10 °C to reflux; (b) sebacic acid (**2**), DIPEA, HATU, CH_2_Cl_2_, r.t.

**Scheme 2:**
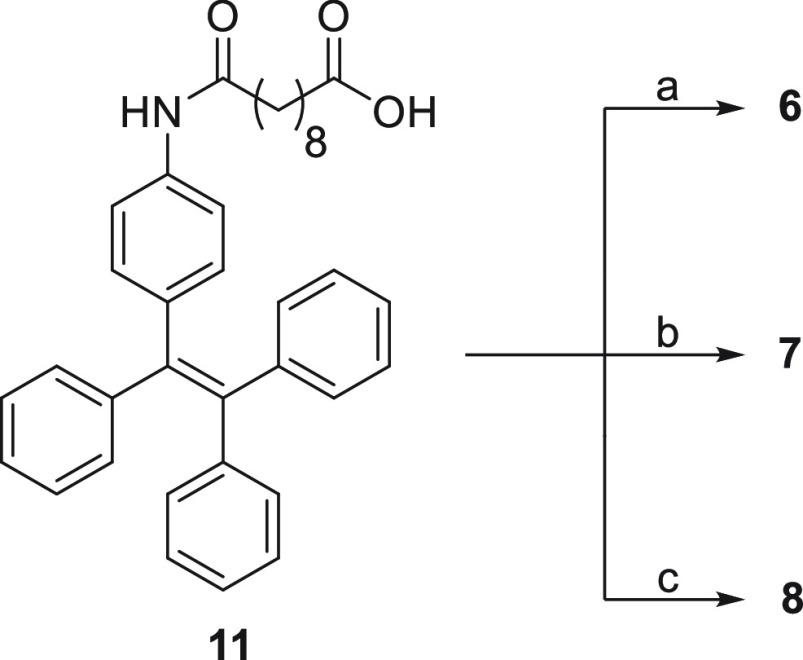
Synthesis of Conjugates **6**–**8** Reagents and conditions: (a) **3**, EDC·HCl, DMAP, CH_2_Cl_2_, r.t., overnight; (b) **4**, HATU, DIPEA, THF, reflux; (c) **5**, EDC·HCl, DMAP, CH_2_Cl_2_, r.t., overnight.

Subsequently, acid **11** was coupled with the appropriate drugs, **3**, **4**, and **5**, in a EDC·HCl/DMAP-mediated condensation reaction for drugs **3** and **5** and HATU/DIPEA-mediated condensation reaction for drug **4** to provide the desired conjugates **6**, **7**, and **8** in good yields (70%, 72%, and 67%, respectively) (Scheme 2). During the synthesis, no unexpected or unusually high safety hazards were encountered.

The successful nanoparticle formation through solvent evaporation protocol of the conjugates **6**, **7** and **8** was evaluated by dynamic light scattering (DLS) measurements (Table 1). All the compounds, formulated at a 2 mg/mL concentration, gave a stable and monodisperse suspension of NPs, characterized by hydrodynamic diameters (HD) in the range of 150–580 nm and a negative ζ -potential lower than −20 mV.

**Table 1:** Hydrodynamic Diameter, Polydispersity Index, and ζ-Potential of Nanoformulated Compounds **6**, **7**, and **8**

compound	hydrodynamic diameter (nm)	polydispersity index (PI)	ζ-potential (mV)
**6-NP**	151.8 ± 1.5	0.104 ± 0.026	–23.92 ± 1.21
**7-NP**	244.1 ± 24.6	0.159 ± 0.014	–33.40 ± 0.30
**8-NP**	574.5 ± 18.8	0.134 ± 0.031	–42.10 ± 0.70

The photoluminescence properties of the nanoparticles dispersed in water were investigated (Table 2 and Figure 3). **6-NP** and **8-NP** showed intense blue fluorescence with quantum yield of 45% and 41%, respectively, and maximum of emission at 480 nm for both, while nanoparticles **7-NP** were not emissive. For comparison, fluorescence emission has been evaluated in diluted organic solution of the conjugates **6**, **7**, and **8** (EtOH for compound **6** and THF for conjugates **7** and **8**). In this case, all of the compounds displayed negligible fluorescence emission, with quantum yield below 1%. This confirmed the expected behavior of TPE-based molecules with AIE character, which are not emissive as isolated molecules in solution and become highly fluorescent when aggregated as NPs.

**Table 2:** Maximum Emission Wavelength (λ_em_) and Quantum Yield (φ_PL_) of Fluorescence from Nanoparticles of Compounds **6**, **7**, and **8** dispersed in water

compound	λ^max^_em_ (nm)	φ_PL_ (%)
**6-NP**	480	45
**7-NP**	475	<1
**8-NP**	480	41

**Figure 3:**
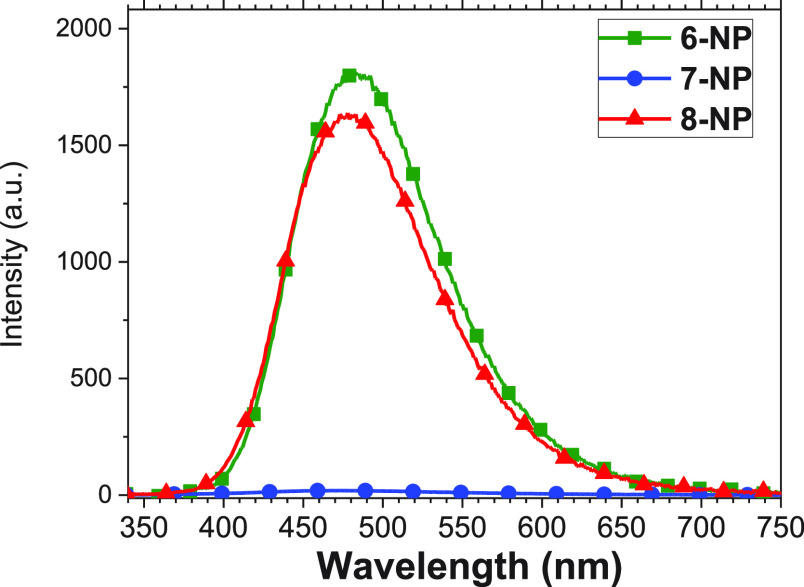
Fluorescence spectra of nanoparticles of conjugates **6**, **7**, and **8** dispersed in water, λ_ex_ = 325 nm.

The antiproliferative activity of the drugs podophyllotoxin (**3**), *N*-desacetylthiocolchicine (**4**), and cabazitaxel (**5**), of conjugates **6**, **7**, and **8**, their corresponding NPs **6-NP**, **7-NP**, and **8-NP**, and of building block **11** was evaluated in three human ovarian (KURAMOCHI, OVCAR3, OVSAHO) and three human breast (MCF7, SKBR 3, and MDA-MB231) cancer cell lines. The results, expressed as IC_50_ values (nM), are presented in Table 3.

**Table 3:** Cell Growth Inhibition (IC_50_) of the Compounds and NPs on Human Cancer Cell Lines

	IC_50_ (μM)^a^					
compound	KURAMOCHI	OVCAR3	OVSAHO	MCF7	SKBR 3	MDA-MB231
**11**	NI^b^	NI^b^	NI^b^	42.8	NI^b^	NI^b^
**3**	0.0011 ± 0.00003	<0.0001	0.0015 ± 0.0001	0.0001 ± 0.000001	0.00003 ± 0.00002	<0.0001
**6**	2.3 ± 0.7	7.1 ± 1.6	45.6 ± 7.5	13.7 ± 0.5	10.4 ± 0.4	17.5 ± 2.8
6-NP	24.3 ± 1.7	8.8 ± 0.3	83.7 ± 6.2	22.5 ± 0.9	40.9 ± 0.3.3	35 ± 2.4
**4**	0.0009 ± 0.0002	<0.0001	0.0037 ± 0.00003	<0.0001	<0.0001	0.2 ± 0.01
**7**	0.7 ± 0.1	2.0 ± 0.1	12.5 ± 1.9	2.0 ± 0.12	1.1 ± 0.5	2.2 ± 0.13
7-NP	0.8 ± 0.07	0.1 ± 0.006	0.4 ± 0.05	6.3 ± 0.1	8.4 ± 0.4	1.7 ± 0.04
**5**	0.0018 ± 0.0002	<0.0001	0.0077 ± 0.001	<0.0001	<0.0001	0.0004 ± 0.0001
**8**	0.0002 ± 0.00006	<0.0001	0.008 ± 0.002	0.0035 ± 0.0002	0.00004 ± 0.00002	0.0002 ± 0.00004
8-NP	0.0393 ± 0.004	0.0007 ± 0.0001	1.065 ± 0.5	0.0073 ± 0.001	0.0013 ± 0.0001	0.0012 ± 0.0004

a IC_50_ values are the concentration of tested agent inducing 50% reduction in cell number compared to control cultures after 72 h of incubation.

b NI = No inhibition.

As expected, podophyllotoxin (**3**), *N*-desacetylthiocolchicine (**4**), and cabazitaxel (**5**) were very effective in inducing cytotoxicity in all cell lines, with IC_50_ values in the nanomolar range. On the contrary, the building block **11** did not show any cytotoxicity in all cell lines except for the MCF7 breast cancer cell line, with activity in the high micromolar range (IC_50_ = 42.8 μM). Interestingly, the antiproliferative effects for both conjugates and their NPs was comparable.

In more detail, podophyllotoxin conjugate **6** and its NP **6-NP** showed cell growth inhibition in the low micromolar range in almost all cell lines, with the highest activity in KURAMOCHI and OVCAR3 cell lines, although lower than the parent drug **3**. A similar effect was observed for the drug *N*-desacetylthiocolchicine (**4**) and the conjugate **7** and its NP **7-NP**, the parental drug being very effective in the very low nanomolar range and the conjugates in the very low micromolar range. Note that the IC_50_ values of **7-NP** were in the nanomolar range in all three ovarian cell lines with the highest activity in the OVCAR3 cell line with an IC_50_ value of 100 nM.

The aforementioned differences in order of magnitude between the drug, the conjugate, and its nanoparticle changed in the case of the drug cabazitaxel (**5**). Now, both the conjugate **8** and its NP **8-NP** showed a biological activity closer to the one of the parent drug in almost all cell lines tested, reaching even the low picomolar range (IC_50_ value of 40 pM for **8** on the SKBR 3 cell line). In the KURAMOCHI and MDA-MB231 the conjugate **8** presented higher activity than that of the parent drug. **8-NP** exhibited its highest activity in the OVCAR3 cell line with an IC_50_ value of 700 pM.

For the nanoparticles that presented fluorescence (**6-NP** and **8-NP**), confocal imaging was carried out to investigate the internalization of the nanoparticles inside the cells. OVCAR3 cells were incubated with **6-NP** and **8-NP** for 24 and 48 h and imaged, showing the presence of the fluorescent nanoparticles inside the cells, as shown in Figure 4.

**Figure 4:**
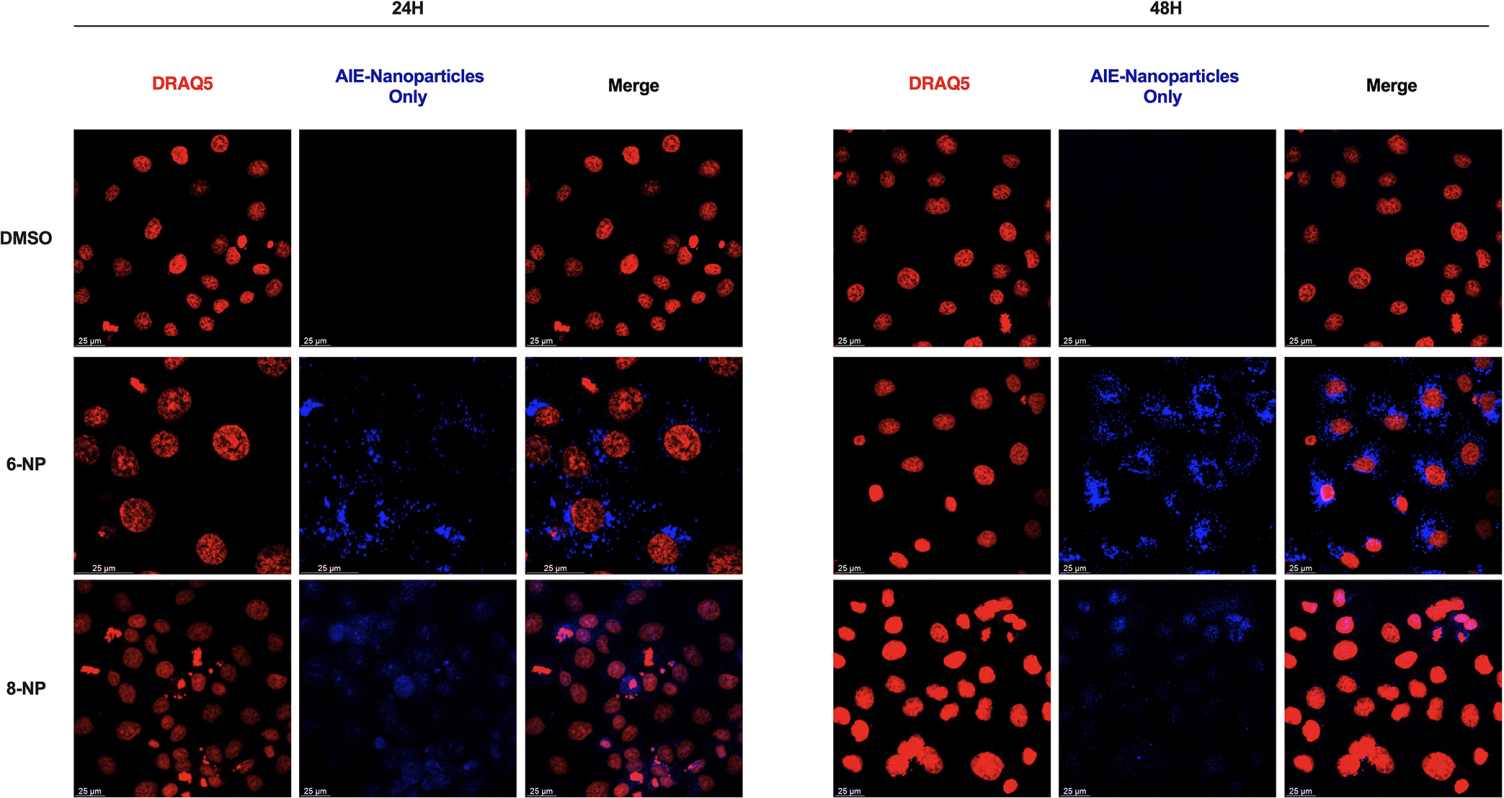
Detection of the internalization of NPs into cells using immunofluorescence assay. Representative images of OVCAR-3 cells after 24 h and 48 h exposure to fluorescent NPs **6-NP** and **8-NP** (Scale bar: 25 μm) (Blue: AEI-Nanoparticles Only, Red: DRAQ5). The nuclei were stained with DRAQ5.

## Conclusions

In this paper, we tested the ability of TPE (**1**) to induce the self-assembly of different types of conjugates containing podophyllotoxin (**3**), *N*-desacetylthiocolchicine (**4**), and cabazitaxel (**5**) as drugs. The formation of NPs was confirmed by dynamic light scattering (DLS) measurements. Moreover, in the case of nanoparticles bearing podophyllotoxin (**6-NP**) and cabazitaxel (**8-NP**), high fluorescence in the blue region was detected as a result of the AIE luminogen character of TPE. The not-emissive character of **7-NP** suggests a role of the drug molecule in the AIE behavior of the nanoassembly and evidence the importance of a careful photophysical investigation of these systems.

The synthesized conjugates (**6** and **7**) and their corresponding NPs (**6-NP** and **7-NP**) were tested for their antiproliferative activity in three human ovarian (KURAMOCHI, OVCAR3, OVSAHO) and three human breast (MCF7, SKBR 3, and MDA-MB231) cancer cell lines. Regarding podophyllotoxin and *N*-desacetylthiocolchicine, the conjugates and their NPs displayed very good antiproliferative activity in the μM range and, in some cases, also in the nanomolar range, but they were less active than the parent drug in both cases. When cabazitaxel was used as a drug, both the conjugate (**8**) and its corresponding NP (**8-NP**) exhibited antiproliferative activity comparable to the parent drug, and in some cases higher than the drug, reaching even low picomolar activity.

The internalization of the fluorescent NPs into the OVCAR-3 cells was investigated using immunofluorescence assay, showing that said nanoparticles were indeed present inside the cells after treatment.

Our results demonstrated that drug conjugate nanoparticles can be obtained by the use of TPE as self-assembly inducer, and this strategy allows maintaining or even enhancing the antiproliferative capacity of the drug, both as a conjugate and as a NP. In addition, TPE can act as AIE luminogen in promoting the emission of the nanoassemblies, making this system extremely interesting for the study of drug delivery and release by fluorescence techniques.

## Supporting Information Available

Experimental details for the synthesis of conjugates, preparation and characterization of nanoparticles and biological evaluation (PDF)

## Abbreviations

AIE: aggregation-induced emission
DIPEA: *N*,*N*-diisopropylethylamine
DMAP: 4-dimethylaminopyridine
EDC·HCl: *N*-ethyl-*N*′-(3-dimethylaminopropyl) carbodiimide hydrochloride
HATU: 1-[bis(dimethylamino) methylene]-1*H*-1,2,3-triazolo[4,5-*b*]pyridinium 3-oxid hexafluorophosphate
NPs: nanoparticles
THF: tetrahydrofuran
TPE: tetraphenylethylene
