# Supplementary material for: Tetraphenylethylene-Based Photoluminescent Self-Assembled Nanoparticles: Preparation and Biological Evaluation
Source: ACS Med Chem Lett. 2023 Sep 29;14(10):1472–7. doi: 10.1021/acsmedchemlett.3c00396 (PMC10577884; doi:10.1021/acsmedchemlett.3c00396)
Supplement: Supplementary file 1 — ml3c00396_si_001.pdf [file ml3c00396_si_001.pdf]

Supporting Information

Tetraphenylethylene-Based Photoluminescent Self-Assembled Nanoparticles: Preparation and Biological Evaluation

Eleonora Colombo,[1,2] Elif Merve Aydın,[3] İdil Su Canıtez,[3] Laura Polito,[4] Marta Penconi,[5] Alberto Bossi,[5] Elisa Impresari,[6] Daniele Passarella,[1] Sabrina Dallavalle,[7] Constantinos M. Athanassopoulos,[8] Sara Pellegrino,[6] Irem Durmaz Şahin,*[9] and Michael S. Christodoulou*[7]

[1]Dipartimento di Chimica, Università degli Studi di Milano, 20133 Milano, Italy

[2]Ann Romney Center for Neurologic Diseases, Department of Neurology, Brigham and Women's Hospital and Harvard Medical School, Boston, MA 02115, USA

[3]Koç University Research Center for Translational Medicine (KUTTAM), Sariyer, Istanbul, Turkey

[4]Istituto di Scienze e Tecnologie Chimiche "Giulio Natta", SCITEC-CNR, 20138, Milano (IT)

[5]Istituto di Scienze e Tecnologie Chimiche "Giulio Natta", SCITEC-CNR, 20138, Milano (IT); SmartMatLab Center, 20133, Milano (IT)

[6]DISFARM, Dipartimento di Scienze Farmaceutiche, Sezione Chimica Generale e Organica "A. Marchesini", Università degli Studi di Milano, Milan, Italy

[7]Department of Food, Environmental and Nutritional Sciences (DeFENS), University of Milan, via Celoria 2, 20133 Milan, Italy

[8]Synthetic Organic Chemistry Laboratory, Department of Chemistry, University of Patras, GR-26504 Patras, Greece

[9]Koç University, School of Medicine, Sarıyer, Istanbul, 34450, Turkey

e-mail: michail.christodoulou@unimi.it, irsahin@ku.edu.tr

Table of contents

General information S2

Synthesis and NMR spectra of compounds **9** – **11** S3

Nanoparticles S9

Fluorescence study S10

Biological assays S11

Chemistry

General information

All reactions were carried out in oven-dried glassware and dry solvents under nitrogen atmosphere. Commercially available reagents and solvents were purchased from Thermo Fischer Scientific or Merck (Sigma Aldrich, Milan, Italy). All tested compounds possessed a purity of > 98% confirmed via elemental analyses (CHN) in a Perkin Elmer 2400 instrument. NMR spectra were recorded on a Brucker Avance 600 MHz and on a Bruker DRX-400 spectrometer employing the residual signal of the deuterated solvent as internal standard. Chemical shifts (δ) are expressed in ppm and coupling constants (J) in Hertz (*Hz*). Merck Silica gel 60 F_{254} (aluminum foil) plates were used for TLC analysis (Sigma Aldrich, Milan, Italy); flash column chromatography was performed on Merck Silica gel (230–400 mesh) (Sigma Aldrich, Milan, Italy). Detection of TLC analyses has been performed under UV light at 254 nm. MS spectra were recorded using electrospray ionization (ESI) technique on a Waters Micromass Q-Tof micro mass spectrometer.

Synthesis

Compound 6.

EDC·HCl (35.0 mg, 0.182 mmol) and DMAP (22.2 mg, 0.182 mmol) were added to a stirred solution of compound **11** (63.0 mg, 0.118 mmol) in dry CH_2Cl_2 (3 mL) under nitrogen atmosphere. After 30 min compound **3** (48.9 mg, 0.118 mmol) was added, and the reaction mixture was left stirring at r.t. overnight. After the reaction completion (TLC monitoring, eluent mixture 1:1 *n*-Hex/EtOAc), 1M HCl (5 mL) was added, and the mixture was extracted with CH_2Cl_2 (3 x 5 mL). The collected organic phases were dried with Na_2SO_4, filtered and concentrated under reduced pressure. The residue was purified by flash chromatography (silica gel, eluent mixture 65:35 *n*-Hex/EtOAc) to obtain the desired product **6** (78.0 mg, 70% yield) as a white amorphous solid.

^{1}H NMR (400 MHz, $CDCl_3$): δ = 7.28 (2H, d, J = 8.3 Hz), 7.22 (1H, s), 7.11 – 7.07 (9H, m), 7.05 – 6.99 (6H, m), 6.96 (2H, d, J = 8.5 Hz), 6.76 (1H, s), 6.54 (1H, s), 6.39 (2H, s), 5.98 (2H, dd, J = 7.1, 1.3 Hz), 5.88 (1H, d, J = 9.1 Hz), 4.61 (1H, d, J = 4.4 Hz), 4.36 (1H, dd, J = 9.3, 7.0 Hz), 4.21 (1H, t, J = 9.9 Hz), 3.80 (3H, s), 3.75 (6H, s), 2.93 (1H, dd, J = 14.5, 4.4 Hz), 2.85 – 2.76 (1H, m), 2.48 – 2.36 (2H, m), 2.24 (2H, t, J = 7.6 Hz), 1.70 – 1.63 (8H, m), 1.35 – 1.24 (8H, m) ppm. ^{13}C-NMR (100 MHz, $CDCl_3$): δ = 174.4, 173.8, 152.8 (2C), 148.4, 147.7, 143.9 (2C), 140.9, 140.4 (2C), 139.6, 137.2, 136.6, 135.0, 132.4, 132.1 (2C), 131.5 (2C), 131.42, 131.39 (4C), 130.4, 129.4, 128.5, 127.9 (2C), 127.7 (4C), 118.8 (2C), 109.8, 108.2 (2C), 107.2, 101.7, 73.5, 60.9, 56.2 (2C), 45.8, 43.8, 39.0, 37.8, 34.5, 29.5, 29.4, 29.29, 29.27, 29.17, 29.12, 27.1, 25.6, 25.2 ppm. MS (ESI), m/z: calcd for $C_{59}H_{61}NO_8$ 911.4, found 912.7 [M+H]+. Anal. Calcd for $C_{59}H_{61}NO_8$: C, 77.69; H, 6.74; N, 1.54. Found: C, 77.81; H, 6.82, N, 1.51.

$[\alpha]_D^{20}$: -555.9° (c = 0.10, $CHCl_3$).

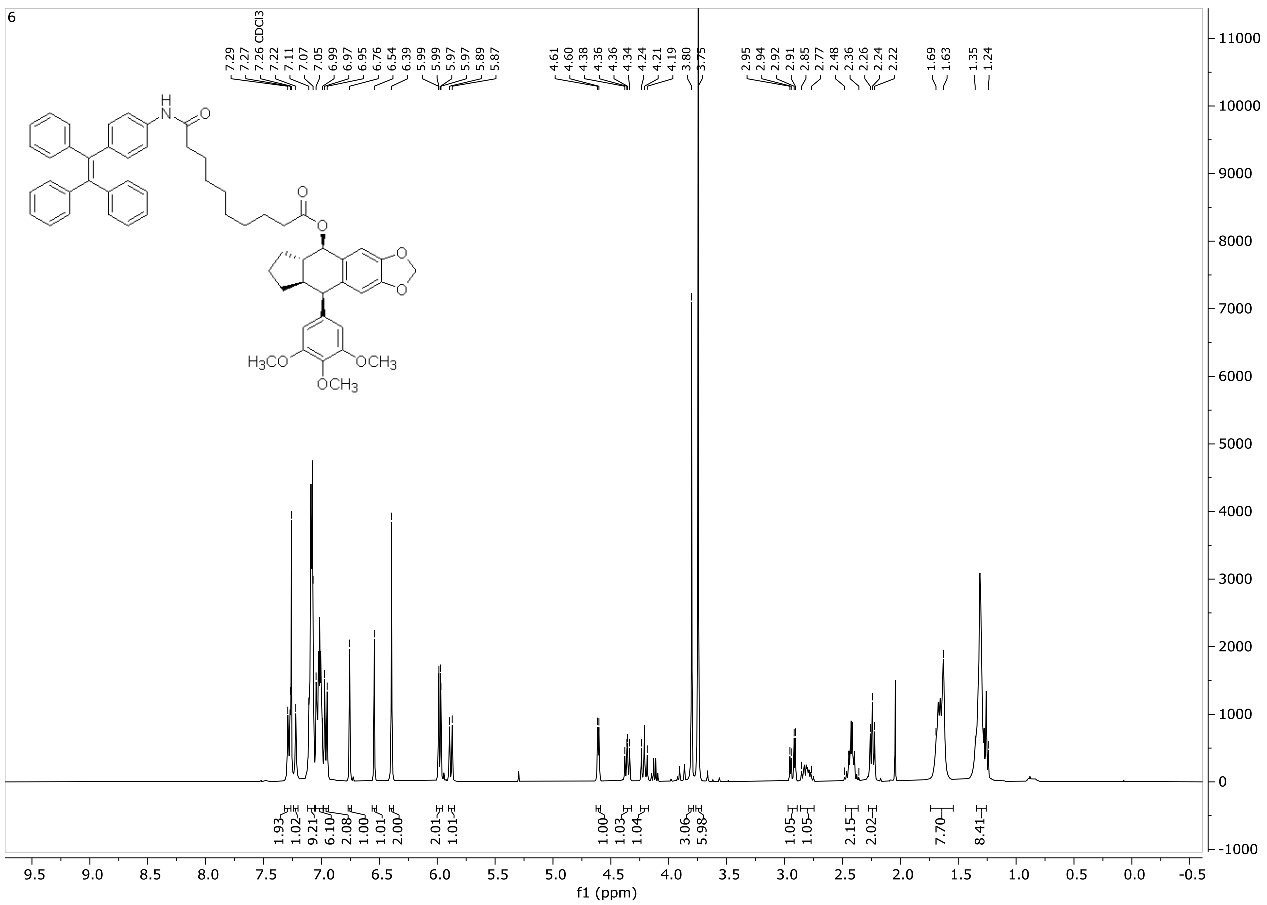

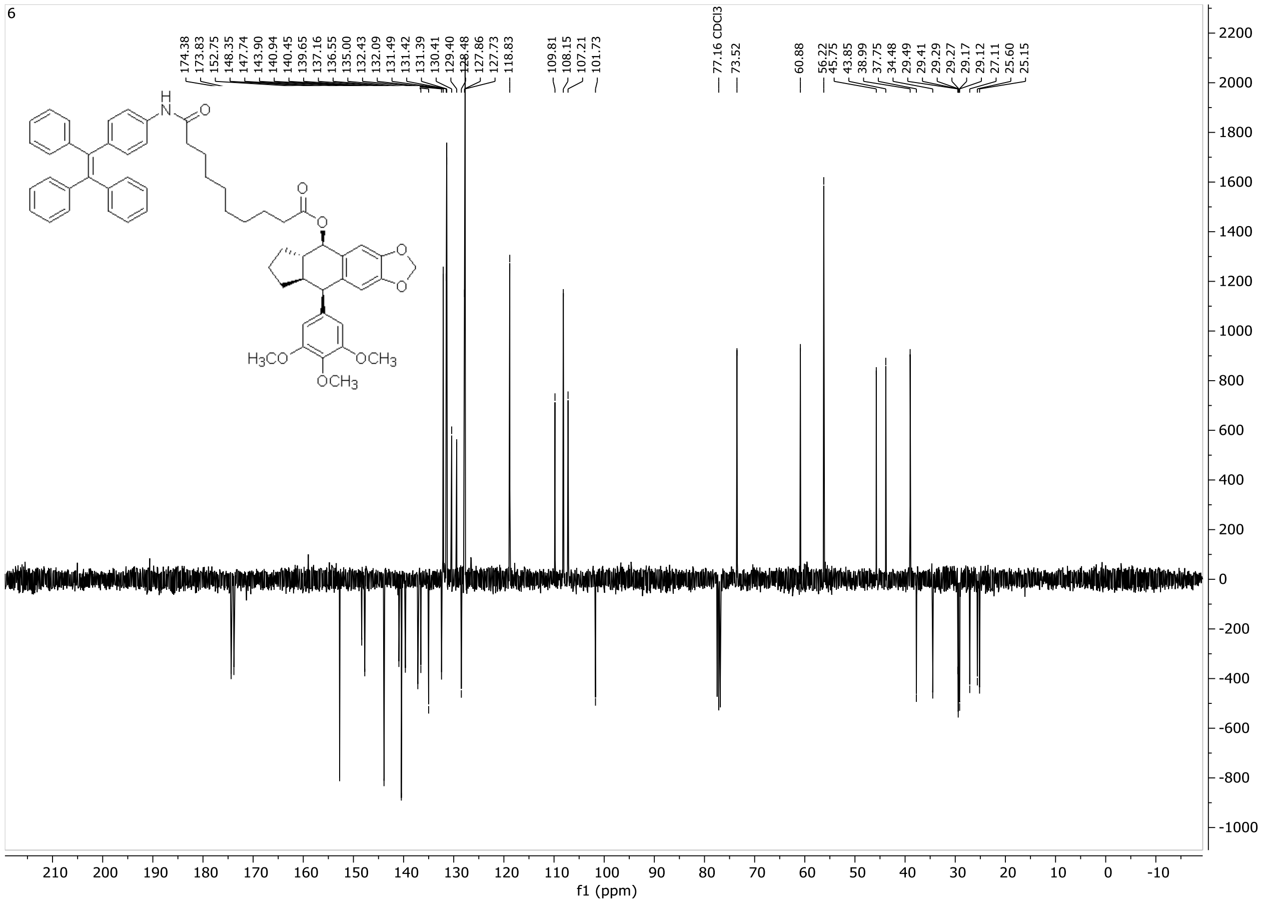

Compound 7.

HATU (39 mg, 0.103 mmol) and DIPEA (0.033 mL, 0.188 mmol) were added to a stirred solution of compound **11** (50 mg, 0.094 mmol) in dry THF (2.4 mL) under nitrogen atmosphere. After 30 min compound **4** (38 mg, 0.103 mmol) was added, and the reaction mixture was reflux for 5h. After the reaction completion (TLC monitoring, eluent mixture 98:2 CH_2Cl_2/MeOH), the solvent was evaporated, CH_2Cl_2 was added and the organic phase was washed with sat. NH_4Cl and brine, dried with Na_2SO_4, filtered and concentrated under reduced pressure. The residue was purified by flash chromatography (silica gel, eluent mixture 98:2 CH_2Cl_2/MeOH) to obtain the desired product **7** (60 mg, 72% yield) as a yellow amorphous solid.

^{1}H-NMR (600 MHz, $CDCl_3$): δ = 8.18 (1H, br. s), 7.55 (1H, s), 7.45 (1H, d, J = 10.2 Hz), 7.37 (2H, d, J = 7.8 Hz), 7.20 (1H, d, J = 10.2 Hz), 7.13 – 7.02 (15H, m), 6.96 (2H, d, J = 8.4 Hz), 6.60 (1H, br. s), 6.57 (1H, s), 4.69 (1H, m), 3.97 (3H, s), 3.94 (3H, s), 3.68 (3H, s), 2.57 – 2.54 (1H, m), 2.47 (3H, s), 2.42 – 2.26 (6H, m), 1.92 (1H, m), 1.75 – 1.59 (4H, m), 1.31 (8H, m) ppm. ^{13}C-NMR (150 MHz, $CDCl_3$): δ = 181.3, 173.1, 172.0, 158.4, 153.9, 151.0, 143.8 (3C), 141.7, 140.5 (2C), 139.0, 137.0, 135.7, 134.4, 131.8, 131.4 (2C), 131.6 (6C), 128.3, 127.7 (3C). 127.6 (6C), 126.4, 126.3, 125.5, 118.8 (2C), 107.5, 61.75, 61.40, 56.17, 52.28, 38.70, 37.37, 36.92, 36.40, 29.98, 29.01, 28.68, 28.52, 25.52, 25.41, 15.62 ppm. MS (ESI), m/z: calcd for $C_{56}H_{58}N_2O_6S$ 887.1, found 888.5 [M+H]+. Anal. Calcd for $C_{56}H_{58}N_2O_6S$: C, 75.82; H, 6.59; N, 3.16. Found: C, 76.08; H, 6.67, N, 3.08. $[\alpha]_D^{20}$: -151.1° (c = 0.10, $CHCl_3$).

7

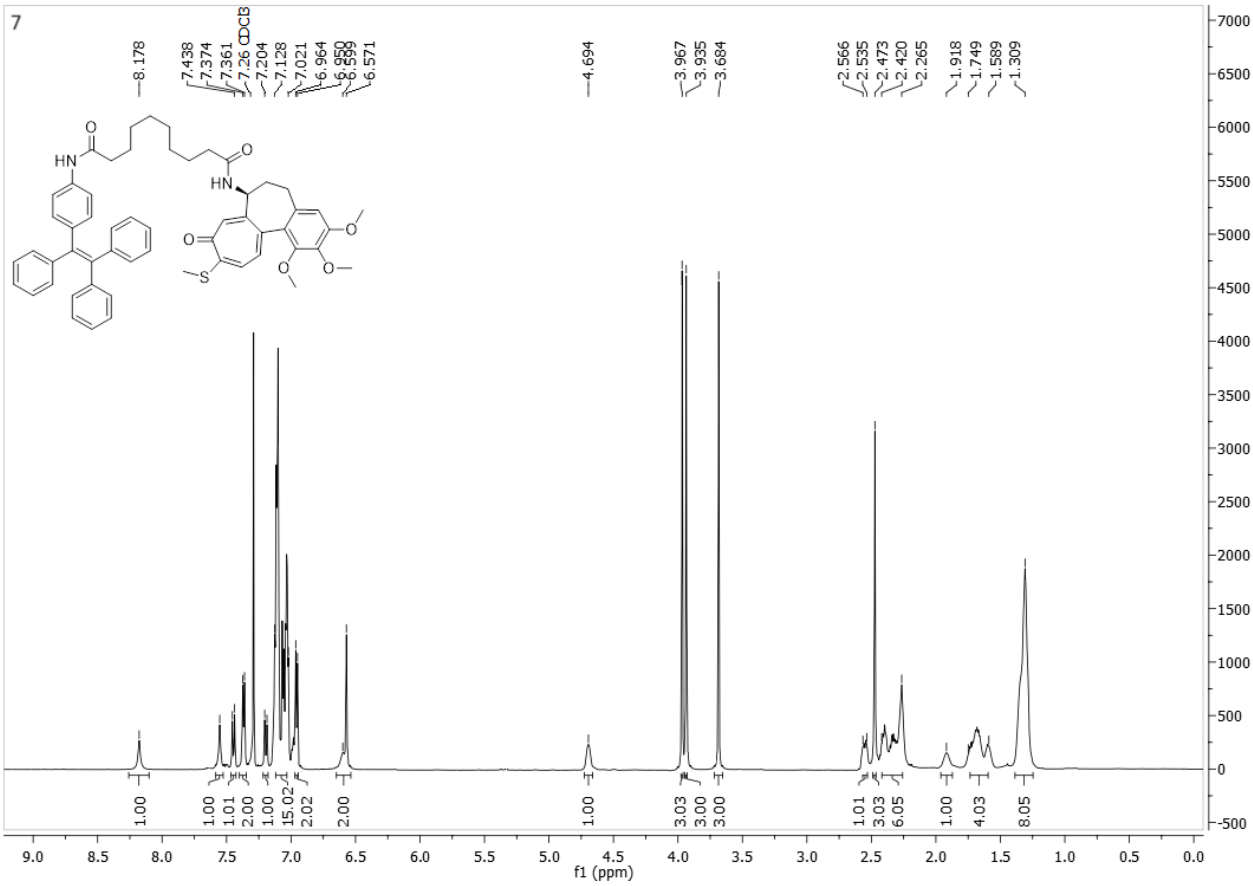
8.178
7.438
7.374
7.361
7.26 CDCl3
7.204
7.128
7.021
6.964
6.950
6.599
6.571
4.694
3.967
3.935
3.684
2.566
2.535
2.473
2.420
2.265
1.918
1.749
1.589
1.309
1.00
1.00
1.01
2.00
1.00
15.02
2.02
2.00
1.00
3.03
3.00
3.00
1.01
3.03
6.05
1.00
4.03
8.05
f1 (ppm)

7

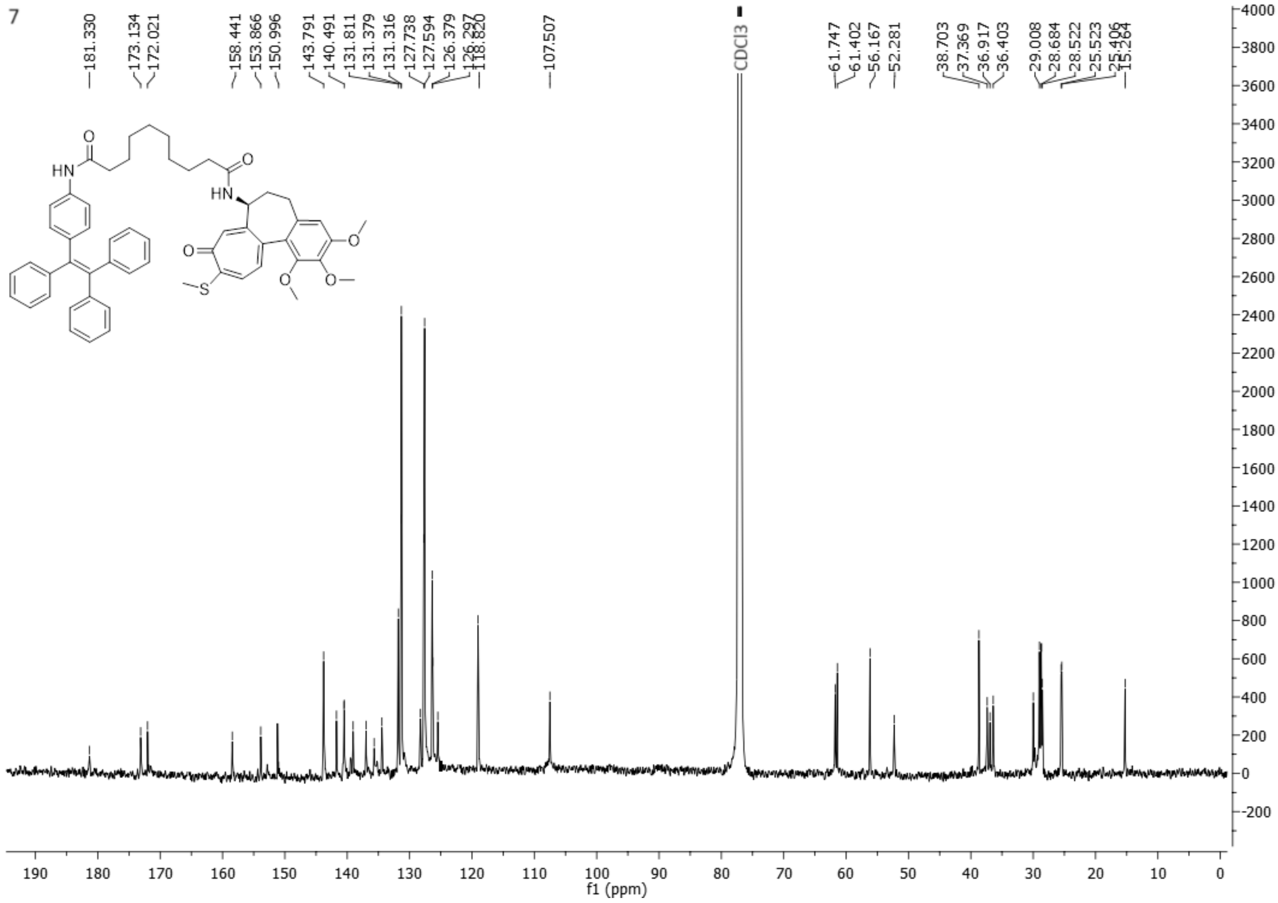
181.330
173.134
172.021
158.441
153.866
150.996
143.791
140.491
131.811
131.379
131.316
127.738
127.594
126.379
126.297
118.820
107.507
CDCl3
61.747
61.402
56.167
52.281
38.703
37.369
36.917
36.403
29.008
28.684
28.522
25.523
25.406
15.264
f1 (ppm)

Compound 8.

EDC·HCl (35.1 mg, 0.188 mmol) and DMAP (23 mg, 0.188 mmol) were added to a stirred solution of compound **11** (50.0 mg, 0.094 mmol) in dry CH_2Cl_2 (3 mL) under nitrogen atmosphere. After 30 min compound **5** (79.3 mg, 0.094 mmol) was added, and the reaction mixture was left stirring at r.t. overnight. After the reaction completion (TLC monitoring, eluent mixture 1:1 *n*-Hex/EtOAc), 1M HCl (5 mL) was added, and the mixture was extracted with CH_2Cl_2 (3 x 5 mL). The collected organic phases were dried with Na_2SO_4, filtered and concentrated under reduced pressure. The residue was purified by flash chromatography (silica gel, eluent mixture 1:1 *n*-Hex/EtOAc) to obtain the desired product **8** (85.2 mg, 67% yield) as a white amorphous solid.

^{1}H NMR (400 MHz, $CDCl_3$): 7.97 (2H, d, *J*=7.4 Hz), 7.46 (1H, t, *J*=7.4 Hz), 7.36 (2H, t, *J*=7.6 Hz), 7.25 (2H, t, *J*=7.5 Hz), 7.15 (5H, dt, *J*=9.9, 5.9 Hz), 7.07 (1H, s), 7.01 – 6.78 (16H, m), 6.12 (1H, t, *J*=8.1 Hz), 5.51 (1H, d, *J*=6.9 Hz), 5.35 – 5.11 (3H, m), 4.86 (1H, d, *J*=9.1 Hz), 4.69 (1H, s), 4.17 (1H, d, *J*=8.4 Hz), 4.03 (1H, d, *J*=8.4 Hz), 3.77 (1H, dd, *J*=10.6, 6.5 Hz), 3.71 (1H, d, *J*=6.8 Hz), 3.29 (3H, s), 3.15 (3H, s), 2.62 – 2.51 (1H, m), 2.37 – 2.10 (7H, m), 2.04 (1H, s), 1.86 (3H, s), 1.76 – 1.47 (8H, m), 1.47 – 1.34 (2H, m), 1.28 – 0.99 (23H, m). ^{13}C-NMR (100 MHz, $CDCl_3$): δ = 205.1, 172.8 (2C), 168.4 (2C), 167.0 (2C), 143.77, 143.75 (2C), 143.6 (2C), 139.7, 138.0, 136.6, 136.3, 135.0, 133.6, 132.0 (2C), 131.4 (2C), 131.33 (2C), 131.31 (2C), 130.2 (2C), 129.3, 128.9 (2C), 128.7 (2C), 128.2, 127.8 (2C), 127.65 (2C), 127.63 (2C), 126.5, 126.42, 126.38, 126.36 (2C), 118.7 (2C), 84.2, 82.6, 81.6, 80.7, 78.9, 77.4, 77.0, 76.7, 74.8, 74.3, 72.0, 57.2, 57.1, 56.8, 47.4, 43.3, 37.7, 35.0, 33.7, 32.0, 29.1, 29.1, 28.9, 28.8, 28.2 (3C), 26.7, 25.4, 24.6, 22.8, 21.1, 14.5, 10.4 ppm. MS (ESI), m/z: calcd for $C_{81}H_{92}N_2O_{16}$ 1348.6, found 1350.7 [M+H]+. Anal. Calcd for $C_{81}H_{92}N_2O_{16}$: C, 72.09; H, 6.87; N, 2.08. Found: C, 72.33; H, 6.95, N, 2.03. $[\alpha]_D^{20}$: -425.9° (*c* = 0.10, $CHCl_3$).

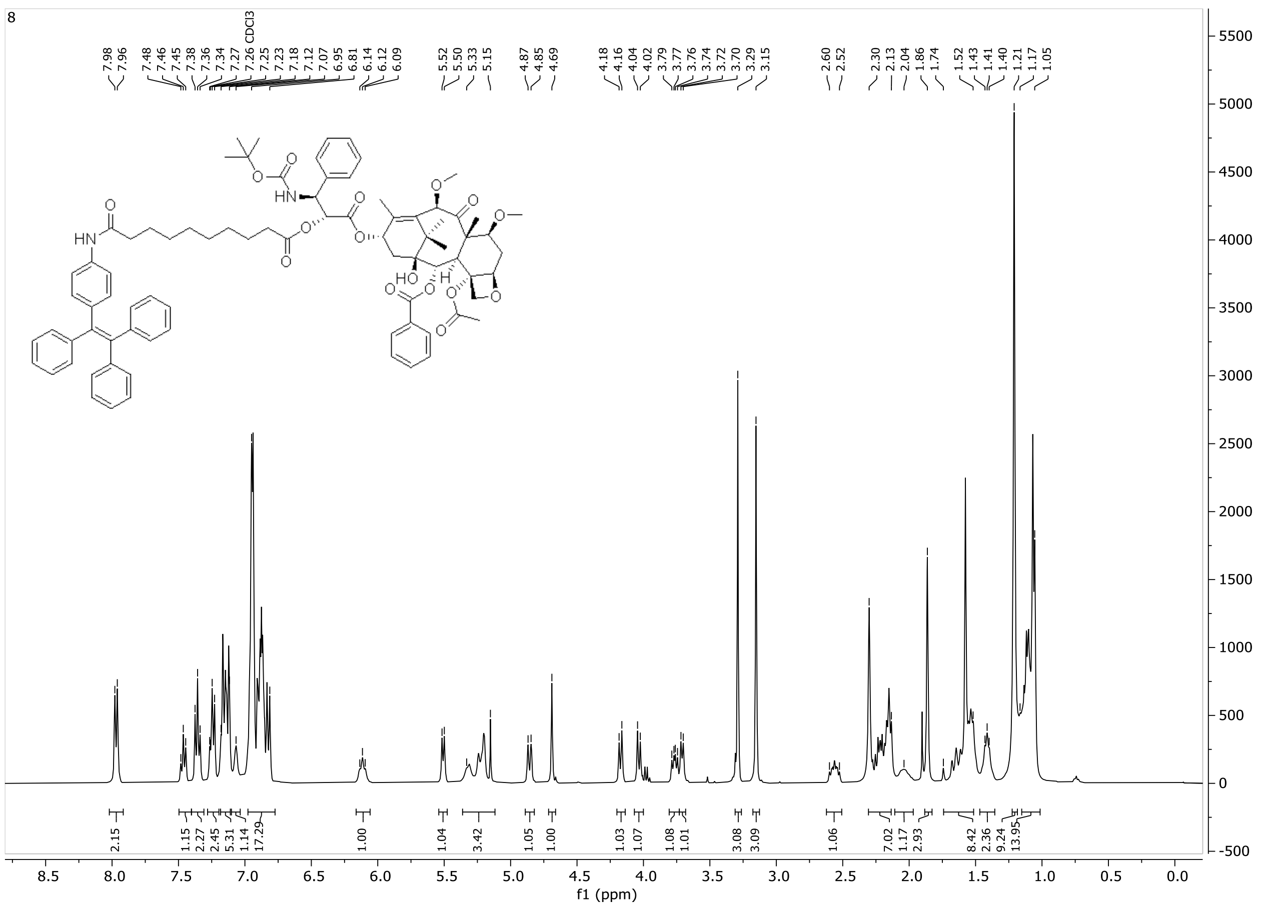

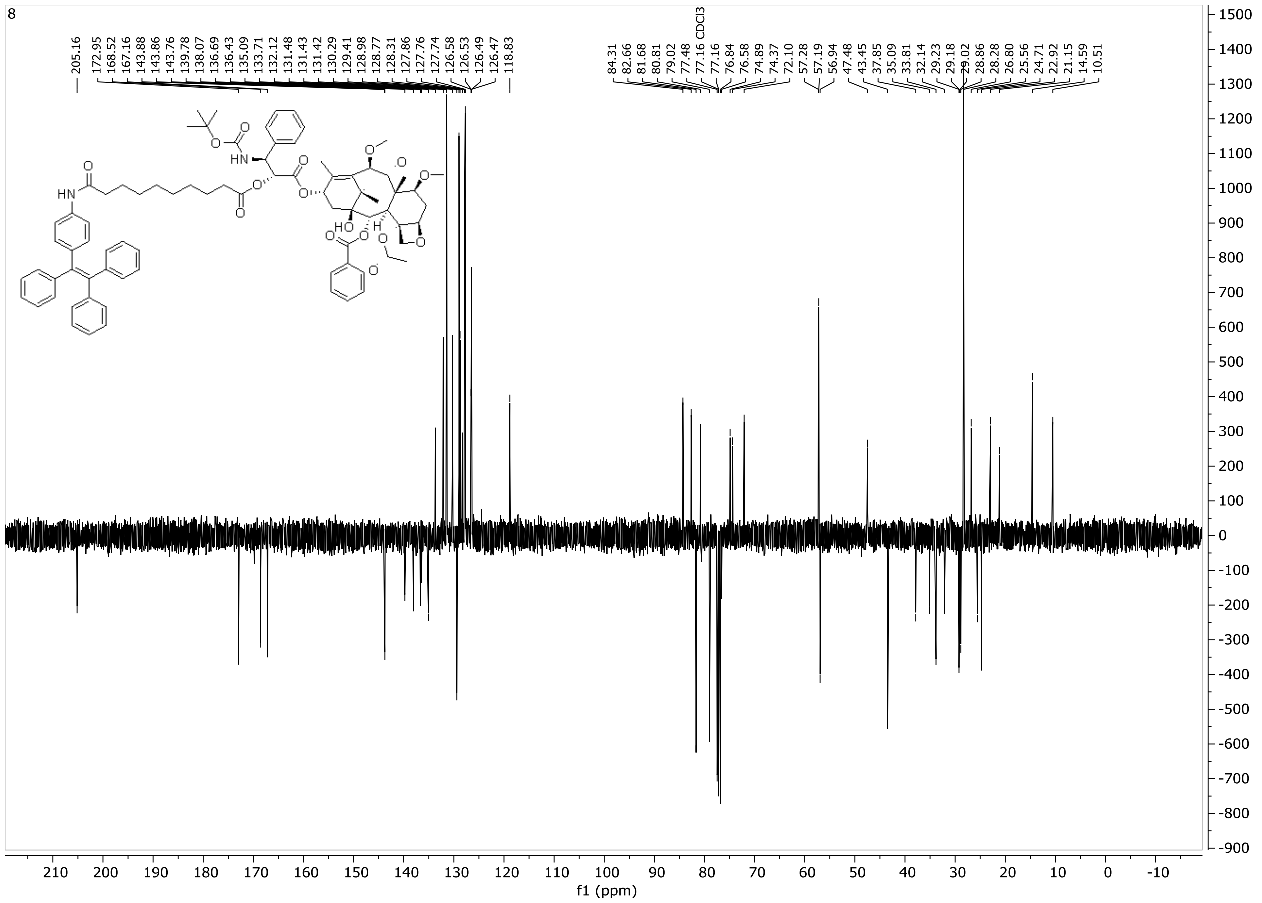

Nanoparticles

Nanoparticles assembly

In accordance with standard solvent evaporation protocols the conjugate (2.0 mg) was first dissolved in the appropriate organic solvent (0.5 mL, THF was used for conjugates **7** and **8**, while EtOH for conjugate **6**) in a vial while stirring at rt. The resulting solution was added dropwise to a round bottom flask containing MilliQ grade distilled water (1 mL) under magnetic stirring (500 rpm). The resulting suspension was stirred for 5 min, then the organic solvent was thoroughly evaporated under reduced pressure, obtaining pure NPs as an opalescent suspension (1 mL, 2 mg/mL).

Nanoparticles characterization

NPs were characterized by dynamic light scattering (DLS), using a 90 Plus Particle Size Analyzer from Brookhaven Instrument Corporation (Holtsville, NY, USA) operating at 15 mW of a solid-state laser (λ = 661 nm), using a 90-degree scattering angle. The ζ-potential was determined at 25 °C using a 90 Plus Particle Size Analyzer from Brookhaven Instrument Corporation (Holtsville, NY, USA) equipped with an AQ-809 electrode, operating at an applied voltage of 120 V. Ten independent measurements of 60 s duration were performed for each sample. Hydrodynamic diameters were calculated using Mie theory, considering the absolute viscosity and refractive index values of the medium to be 0.890 cP and 1.33, respectively. The same aqueous samples at a concentration of 0.2 mg/mL were used for ζ-potential measurement, without any change for the ionic strength (no addition of KCl). The ζ-potential was calculated from the electrophoretic mobility of nanoparticles, by using the Smoluchowski theory.

Fluorescence study

UV–vis absorption spectra were obtained on a Shimadzu UV–vis–NIR 3600 spectrophotometer in a 1 cm path length quartz cell. Photoluminescence quantum yields were measured with a C11347 Quantaurus-QY absolute photoluminescence quantum yield spectrometer (Hamamatsu Photonics), equipped with a 150W xenon lamp, an integrating sphere, and a multi-channel detector. Emission spectra were obtained with an FLS 980 spectrofluorimeter (Edinburg Instrument Ltd.) under continuous excitation provided by a 450 W xenon arc lamp and corrected for detector sensitivity. Photoluminescence experiments were carried out at room temperature in air equilibrated medium. Absorption and emission measurements on nanoparticles were conducted after dilution of the suspension with MilliQ grade distilled water to reduce light scattering. Experiments in homogeneous solution were carried out in ethanol for conjugate **6** and in tetrahydrofuran for conjugates **7** and **8**, at concentration between 2×10^{-5} M and 4×10^{-6}M, no fluorescence emission was detected.

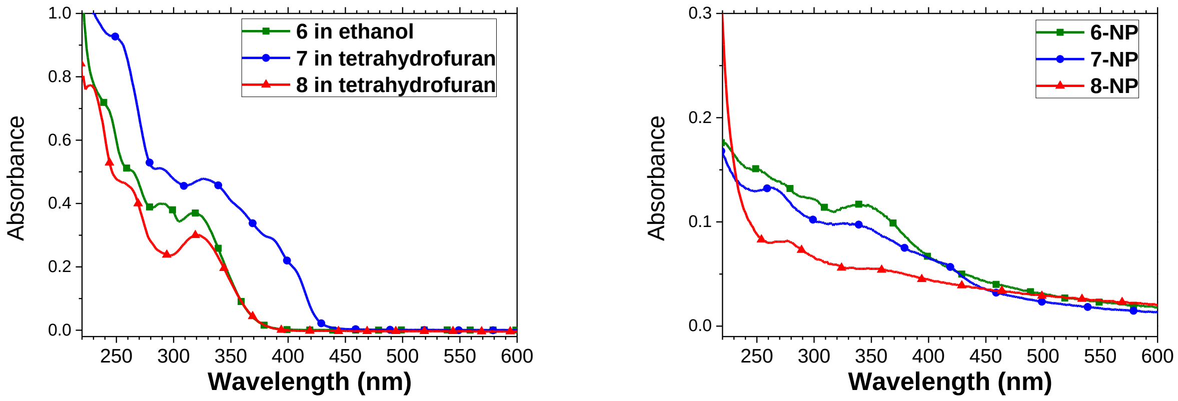

Absorption spectra of conjugates in diluted organic solution (left) and as dispersed nanoparticles in water (right).

Confocal Imaging of NPs

Ovarian cancer cell line, OVCAR-3, were cultured in 24-well plates on coverslips at 1×10^5 cells/well. 24 hours later, growth medium was changed with fresh medium containing the NPs of 6 and 8 conjugates at IC_{75} concentration or medium control for 24 and 48 hours. Cells were fixed with 4% Paraformaldehyde for 20 minutes. Fixed cells were permeabilized for 15 minutes at RT, dark with 0.1% TritonX-100 in 1X DPBS. The cells were washed once with 0.1% Tween-20 in 1x DPBS, and blocked with SuperBlock (ScyTek Laboratories) for 20 minutes at RT. Coverslips were placed on 17.5 µl of the DRAQ5™ Fluorescent Probe Solution (10 µM working solution) (Thermo Fischer, #62254), which stained nuclei as red and incubated for 20 minutes at RT. 6 ul of Mounting Medium was placed on Thermo SuperFrost microscope slides (Thermo Fischer). The coverslips incubated

with DRAQ5 probe were immersed into washing buffer to remove excess antibody solution and then placed on mounting medium by sealing with nail polish to stabilize the coverslips and visualized by confocal microscope (Leica DMi8 /TCS SP8-DLS). The results obtained by n=2 independent experiments.

Biological assays

Cell Culture

OVCAR3, OVSAHO and KURAMOCHI high grade serous ovarian cancer cell lines were grown in RPMI 1640 medium containing 1% penicillin/streptomycin, 1% non-essential amino acids and 10% fetal bovine serum (FBS); MDA-MB231, SK-BR-3 and MCF-7 breast cancer cell lines were grown in DMEM high glucose medium containing 1% penicillin/streptomycin, 1% non-essential amino acids and 10% fetal bovine serum (FBS) and incubated at 37 ºC with 5% CO_2.

Cytotoxicity screening with NCI-Sulforhodamine B assay.

OVCAR3, OVSAHO and KURAMOCHI high grade serous ovarian cancer cell lines and MDA-MB231, SK-BR-3 and MCF-7 breast cancer cell lines were inoculated into 96-well plates as 3000 cells/well. After 24h incubation, cells were treated with the compounds in increasing concentrations (0.078 µM – 40 µM). Each and every drug treatment was performed in triplicate. DMSO was used as negative control. After 72h of incubation time, medium was discarded and plates were washed twice with 1xPBS. Then the cells were fixed with 10% (w/v) trichloroacetic acid (TCA) solution for 1h in dark at +4 °C. In order to remove TCA, cells were then washed with ddH_2O about 4-5 times and left air-dry at room temperature. The plates were then stained using 0.4% sulforhodamine B (SRB) solution in 1% acetic acid and incubated in dark at room temperature for 10min. Finally, excess dye was discarded by washing off by 1% acetic acid 4-5 times until no dye comes out and left air dry at room temperature. Lastly, 10 mM cold TBS solution was used to solubilize the protein-bound SRB dye. Absorbance values were measured at 515nm with microplate reader. In order to calculate IC_{50} values, the recorded OD value for each well was normalized to the OD value of its corresponding DMSO control.
